# Theranostic Designed Near-Infrared Fluorescent Poly (Lactic-co-Glycolic Acid) Nanoparticles and Preliminary Studies with Functionalized VEGF-Nanoparticles

**DOI:** 10.3390/jcm9061750

**Published:** 2020-06-05

**Authors:** Michela Varani, Filippo Galli, Gabriela Capriotti, Maurizio Mattei, Rosella Cicconi, Giuseppe Campagna, Francesco Panzuto, Alberto Signore

**Affiliations:** 1 Nuclear Medicine Unit, Department of Medical-Surgical Sciences and of Translational Medicine, Faculty of Medicine and Psychology, “Sapienza” University of Rome, 00189 Roma, Italy; varanimichela@gmail.com (M.V.); filippo.galli@uniroma1.it (F.G.); gabriela.capriotti@uniroma1.it (G.C.); gius.campagna@gmail.com (G.C.); 2 Department of Biology and Centro di Servizi Interdipartimentale-Stazione per la Tecnologia Animale, “Tor Vergata” University of Rome, 00189 Roma, Italy; mattei@uniroma2.it (M.M.); rosella.cicconi@uniroma2.it (R.C.); 3 Digestive Disease Unit, Sant’Andrea University Hospital, ENETS Center of Excellence Rome, 00189 Roma, Italy; fpanzuto@ospedalesantandrea.it

**Keywords:** polymeric nanoparticles, PLGA, optical imaging, tumor targeting

## Abstract

Poly-lactic-co-glycolic acid nanoparticles (PLGA-NPs) were approved by the Food and Drug Administration (FDA) for drug delivery in cancer. The enhanced permeability and retention (EPR) effect drives their accumulation minimizing the side effects of chemotherapeutics. Our aim was to develop a new theranostic tool for cancer diagnosis and therapy based on PLGA-NPs and to evaluate the added value of vascular endothelial growth factor (VEGF) for enhanced tumor targeting. In vitro and in vivo properties of PLGA-NPs were tested and compared with VEGF-PLGA-NPs. Dynamic light scattering (DLS) was performed to evaluate the particle size, polydispersity index (PDI), and zeta potential of both preparations. Spectroscopy was used to confirm the absorption spectra in the near-infrared (NIR). In vivo, in BALB/c mice bearing a syngeneic tumor in the right thigh, intravenously injected PLGA-NPs showed a high target-to-muscle ratio (4.2 T/M at 24 h post-injection) that increased over time, with a maximum uptake at 72 h and a retention of the NPs up to 240 h. VEGF-PLGA-NPs accumulated in tumors 1.75 times more than PLGA-NPs with a tumor-to-muscle ratio of 7.90 ± 1.61 (versus 4.49 ± 0.54 of PLGA-NPs). Our study highlights the tumor-targeting potential of PLGA-NPs for diagnostic and therapeutic applications. Such NPs can be conjugated with proteins such as VEGF to increase accumulation in tumor lesions.

## 1. Introduction

The latest advances in molecular imaging are closely related to the use of new tools, such as nano- or micro-particles that can be used for several applications, from detection and diagnosis to drug delivery and treatment [1]. Different nanomaterials are used to create particles with a range from 1 to 1000 nm, and so, are defined as nano-particles (NPs) [2]. They offer the advantage to deliver drugs to the target with high efficiency and low systemic toxicity [3,4]. The NPs formulated with organic polymers (polymeric NPs) are generally one of the best choices for clinical or pre-clinical use due to their favorable characteristics such as non-immunogenicity, non-toxicity, biodegradability, and biocompatibility [5]. Indeed, PLGA have been approved by the Food and Drug Administration (FDA) and the European Medicine Agency (EMA) as copolymers to deliver drugs, and today about 16 approved pharmaceuticals are based on the use of these NPs [6,7]. Their in vivo biodistribution is greatly influenced by different physical and chemical characteristics, among which size and glycolic:lactic acid ratio play a key role [8,9]. The NPs accumulate in target lesions with an active or passive mechanism. The passive mechanism is represented by the enhanced permeability and retention (EPR) effect, that allows NPs with a size in the 20–200 nm range to accumulate in cancer lesions with an impaired vasculature [10,11]. Therefore, this mechanism is of great importance when using nanotechnologies in oncology [12,13,14]. Moreover, the flexibility of PLGA-NPs offers the advantage to combine their ability to deliver drugs with the possibility to functionalize them with peptides, proteins, or imaging probes [15]. Since tumor and stromal cells produce several proangiogenic factors, such as proteins from the vascular endothelial growth factor (VEGF) family, they are usually characterized by high and irregular vascularization [16]. Therefore, targeting of either VEGF or VEGF receptor (VEGFR) can be achieved and exploited to increase PLGA accumulation in tumor lesions [17].

In the present study, we investigated the possibility of using specifically designed PLGA-NPs as a tool for future theranostic applications. We selected PLGA-NPs (lactic acid:glycolic acid ratio of 50:50, average size of 100–200 nm) conjugated with a near-infrared (NIR) fluorochrome with an excitation wavelength at 780 nm and emission wavelength at 825 nm, allowing a deeper tissue penetration of fluorescence [18]. The target capacity and the pharmacokinetic of native PLGA-NPs was investigated in vivo to evaluate the tumor detection and then the retention of PLGA-NPs up to 240 h.

To actively target tumor cells over-expressing VEGFR, the PLGA-NPs were loaded with a recombinant human VEGF-A165 (rhVEGF) analog by the 1-Ethyl-3-[3-dimethylaminopropyl] carbodiimide hydrochloride/N-hydroxysuccinimide (EDC/NHS) covalent coupling method. The successful functionalization of NPs was examined with an in vitro kinetic binding of VEGF-PLGA-NPs with the VEGF Receptor-2 (KDR)/Fc chimera human compared to native PLGA-NPs. Tumor targeting of VEGF-PLGA-NPs was examined in vivo 24 h post-injection (p.i.) and compared with native PLGA-NPs. The T/M showed an increasing of PLGA-NPs capability to target the tumor over-expressing VEGFR.

## 2. Materials and Methods

### 2.1. PLGA-NPs

PLGA (D, L-lactide-co-glycolide) nanoparticles with a lactic acid:glycolic acid ratio of 50:50, average size of 100–200 nm, conjugated with a NIR fluorochrome with an excitation/emission wavelength of 780/825 nm, were purchased from Degradex^®^ (Phosphorex Inc., Hopkinton, MA, USA).

### 2.2. PLGA-NPs Functionalization with VEGF

The conjugation of VEGF was performed by using the 1-Ethyl-3-(3-dimethylaminopropyl) carbodiimide (EDC) and N-hydroxysuccinimide (NHS) coupling protocol. EDC and NHS were purchased from Thermo Scientific (ThermoFisher, Waltham, MA, USA). The recombinant human VEGF-A165 analog with a molecular weight of 38.2 kDa was purchased by Prospec-Tany Technogene Ltd. (Rehovot, Israel). This molecule shares 88% homology with murine VEGF and has been previously used in mice [19,20]. The carboxylate (-COOH) PLGA-NPs react with NHS in the presence of EDC to create a stable crosslinking with the primary amines (-NH_2_) of the VEGF molecule. The conjugation condition was initially optimized with the use of bovine serum albumin (BSA), evaluating the protein-particle ratio, pH, the choice of buffer, the reaction time, and the purification method. A suspension of 6 mL MES buffer (pH 6.0) containing a concentration of PLGA-NPs (5 mg/mL) were first reacted with 30 mg of EDC (5 mg/mL in phosphate-buffered saline (PBS) pH 7.4). Then, 30 mg of NHS (5 mg/mL in PBS, pH 7.4) were added to the solution and incubated at room temperature with agitation for 15 min. To separate the activated PLGA-NPs from an excess of EDC, EDC-by-products, and NHS, the sample was centrifuged with a high-speed micro-centrifuge (ThermoFisher, Waltham, MA, USA) at 12,000 rpm (9500× *g*) 4 °C for 20 min and washing 3 times with 1 mL PBS (pH 7.4). The EDC coupling creates an unstable reactive o-acylisourea ester group that is easily substituted by an amine-reactive ester in the presence of NHS. The resulting NHS ester is semi-stable but very reactive towards the amino groups on the VEGF molecule. The carboxyl-amine reaction allows the conjugation of the VEGF onto the PLGA-NPs. RhVEGF (2 mg) was added to the PLGA-NPs suspension and the conjugation proceeded for 2 h at room temperature. The resulting VEGF-PLGA-NPs were collected by 3 times ultracentrifugation at 12,000 rpm (9500× *g*), 4 °C for 20 min, and was washed with 1 mL of PBS (pH 7.4) to remove unconjugated VEGF.

### 2.3. Calculation of Average Size and Zeta Potential

The mean size, the polydispersity index (PDI), and the net surface charges (zeta potential) of native and functionalized PLGA-NPs were measured by dynamic light scattering (DLS), using photon correction spectroscopy, electrophoretic mobility analysis, and potential distribution at 25 °C with water as suspension medium. Reading was performed with a NanoZetaSizer analyzer (Malvern Instruments Ltd., Malvern, UK) equipped with a 5 mW HeNe laser (wavelength *λ* = 632.8 nm), a digital logarithmic correlator and a non-invasive backscattering (NIBS) optical system. Briefly, 10 µL (100 µg) PLGA-NPs were suspended with 90 µL H_2_O and loaded in Sarstedt polystyrol/polystyrene cuvettes (10 × 10 × 45 mm) for size and PDI measurements. For zeta potential analysis, 20 µL (200 µg) PLGA-NPs and VEGF-PLGA-NPs were suspended with 980 µL H_2_O, sonicated to reduce the aggregation and loaded in Malvern folded capillary cells for zeta potential measurements. Absorption spectra were acquired by a Jasco V-630 spectrophotometer. Briefly, 50 µL (500 µg) PLGA-NPs and VEGF-PLGA-NPs were diluted with 400 μL H_2_O and loaded in J18 Jasco quartz cells (path length = 10 mm). Water solution was measured separately as a blank solution and subtracted by sample spectra. All experiments were performed in triplicate.

### 2.4. In Vitro Binding of VEGF-PLGA-NPs and PLGA-NPs to KDR-Fc

In vitro binding of native or VEGF-functionalized PLGA-NPs was performed with Nunc MaxiSorp™ 96 well plates (ThermoFisher, Waltham, MA, USA). The binding properties due to the hydrophilic surface of the wells allowed the coating of the VEGF Receptor-2 (KDR)/Fc chimera human (Sigma-Aldrich, St. Louis, MO, USA). Briefly, 50 µL of KDR-Fc in a final concentration of 0.002 µg/µL in bicarbonate/carbonate coating buffer (100 mM) was added to each well and the plate was covered and incubated 48 h at 4 °C. The coating solution was removed and the wells were rinsed two times with PBS (pH 7.4). Then, 150 µL of skimmed milk powder 2% (*w/v*) in PBS were added per well to block residual binding sites for 1 h at 37 °C. As a negative control, a blocking solution was added to each well that had not been coated with KDR-Fc. The blocking solution was removed by rinsing twice with 1 × PBS, pH 7.4. Then, 100 µL of two-fold dilution of VEGF-PLGA-NPs and native PLGA-NPs were added to each well followed by overnight incubation at 4 °C. KDR-Fc-uncoated wells were used to evaluate non-specific binding to the plastic. After incubation, the plate was washed two times with PBS and imaged with an in vivo FX station (Molecular Imaging Software, Kodak, Sevier County, TN, USA). Regions of interest (ROIs) were drawn for each well and the mean fluorescent intensity (mean IF) was calculated. The mean IF from wells without KDR-Fc (−KDR) was subtracted to the mean IF calculated in well coated with KDR-Fc (+KDR) to obtain PLGA-NPs and VEGF-PLGA-NPs net binding to KDR-Fc. Experiments were performed in triplicate.

### 2.5. In Vivo Studies

#### 2.5.1. Mouse Model

All animal experiments were carried out in compliance with the local ethics committee and in agreement with the National rules and the EU regulation (Study 204/2018-PR). A syngeneic murine tumor model was used for in vivo studies. The model was obtained by subcutaneous injection in the right thigh of 10^6^ J774a.1 cells (reticulum cell sarcoma) in a medium: Matrigel^®^ (BD-Biosciences, Bergen, NJ, USA) solution (200 μL, 50:50, v:v), in female BALB/c mice (8 weeks). Cells were purchased from American Type Culture Collection (ATCC^®^ TIB-67™, Milan, Italy) and grown in ATCC-formulated Dulbecco’s Modified Eagle’s Medium supplemented with 10% of fetal bovine serum at 37 °Cand in 5% CO_2_. The 8-week-old female BALB/c mice were purchased from Harlan Laboratories. After about 20 days from the inoculation, the tumors became palpable and the targeting experiments were performed.

#### 2.5.2. Pharmacokinetic of PLGA-NPs

To evaluate the kinetics and tumor targeting of native PLGA-NPs, 100 μL of NPs (500 μg) diluted with 50 μL NaCl were injected in the tail vein of 22 BALB/c mice, bearing a subcutaneous syngeneic tumor (reticulum cells sarcoma). At 2, 24, 48, and 72 h p.i. 5 mice per time point were anesthetized to acquire whole-body images with a Kodak in vivo FX station. Then, mice were sacrificed to excise major organs (liver, spleen, lungs, kidneys, muscle, tumor) to perform ex vivo optical imaging and quantify the uptake of PLGA-NPs in selected organs. ROIs were drawn over each organ, and the fluorescence signal was calculated as net fluorescence/area of the organ. Whole-body optical imaging only was performed in the last two mice up to 240 h.

#### 2.5.3. Tumor Targeting of VEGF-PLGA-NPs and of PLGA-NPs

For tumor targeting experiments, fluorescent PLGA-NPs and VEGF-PLGA-NPs (500 µg in 150 μL 0.9% NaCl solution) were injected in the tail vein of 10 BALB/c mice (5 mice for each compound), bearing a subcutaneous syngeneic tumor (reticulum cells sarcoma). After 24 h, whole-body images were acquired and then mice were sacrificed. Liver, spleen, lungs, kidney, muscle, and tumor were excised for ex vivo optical imaging. On ex vivo images, ROIs were drawn to quantify the uptake of PLGA-NPs and VEGF-PLGA-NPs in selected organs and to calculate the tumor-to-muscle ratio (T/M). The fluorescence signal was calculated as net fluorescence/area.

### 2.6. Statistical Analysis

Statistical analysis was performed using SAS v. 9.4 (SAS Institute Inc., Cary, NC, USA).

Variables continuous was showed as mean ± standard deviation (SD). Shapiro-Wilk test was used to verify the normality of distribution of continuous variables. We applied the Box-Cox procedure which allowed to identify suitable mathematical functions (log10, quadratic, and inverse) which make the non-normal continuous variables/residuals subsequently distributed according to the Gauss condition. Comparisons between “PLGA-NPs” vs. “VEGF-PLGA-NPs” and continuous variables were analyzed by *t*-test. We used the Satterthwaite formula when the variances were unequal. Differences between time (2 h, 24 h, 48 h, and 72 h) and the continuous variables were tested by GLM (General Liner Model) test. Homoscedasticity was verified by Levene and Brown-Forsythe tests. Post-hoc analysis was performed by the Tukey test. Mann-Whitney test was used comparing data of net binding of PLGA-NPs and VEGF-PLGA-NPs to KDR. A *p* < 0.05 was considered statistically significant.

## 3. Results

### 3.1. Characterization of Native and VEGF Functionalized PLGA-NPs

**Figure 1:**
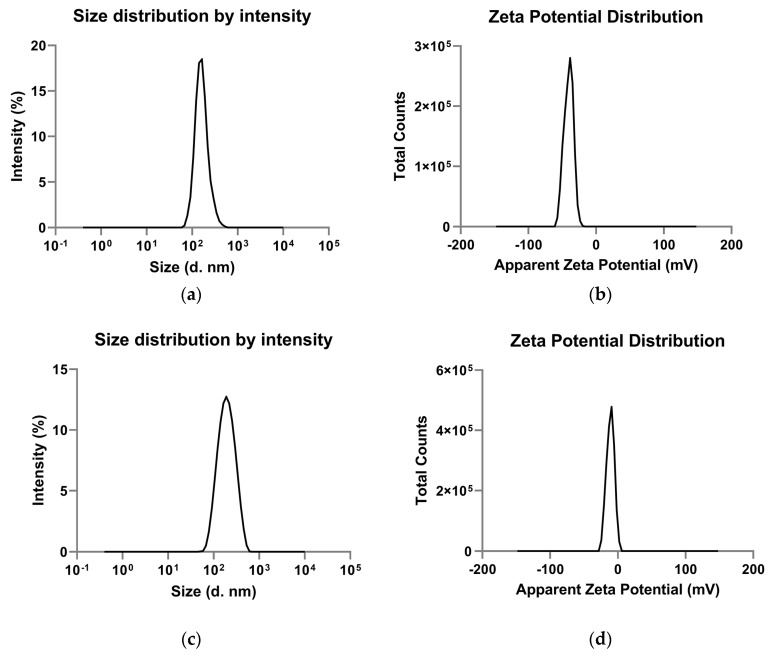
Particle size distribution and zeta measurement profile of native (**a,b**) and vascular endothelial growth factor-conjugated poly-lactic-co-glycolic acid nanoparticles (VEGF-PLGA-NPs) (**c,d**). Data are three different measurements made by the instrument on the same sample. (**a**) Size distribution of PLGA-NPs; (**b**) zeta potential distribution of PLGA-NPs; (**c**) size distribution of VEGF-PLGA-NPs; (**d**) zeta potential distribution of VEGF-PLGA-NPs.

Preliminary characterization showed that native PLGA-NPs have a zeta average of 180 ± 17.08 nm with a PDI of 0.25 ± 0.02. The VEGF-PLGA-NPs have a zeta average of 173 ± 7.39 nm with a PDI of 0.17 ± 0.01. The zeta average and the PDI were reported as mean ± standard deviation (SD) of three measurements performed on the same sample (Table 1). Native PLGA-NPs showed a negative zeta potential value of −37.60 ± 0.67 mV, that excludes the presence of aggregates due to the Van der Waal interactions. The VEGF-PLGA-NPs have a zeta potential value of −9.43 mV that indicated a change in the potential difference across the boundaries between liquid and the NPs surface, revealing that the conjugation was successful (Figure 1).

**Figure 2:**
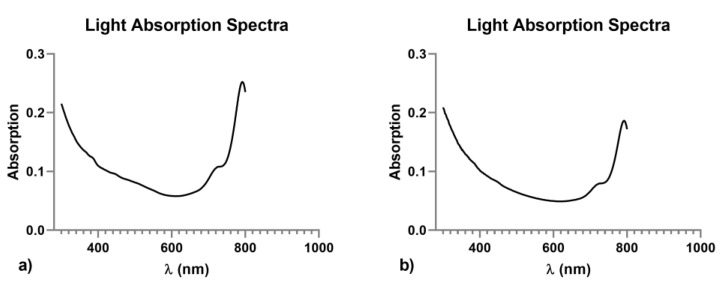
Light absorption spectra of poly-lactic-co-glycolic acid nanoparticles (PLGA-NPs) (**a**), and vascular endothelial growth factor-conjugated poly-lactic-co-glycolic acid nanoparticles (VEGF-PLGA-NPs) (**b**). Both the nano-formulations generated an emission peak >700 nm. Solvent was bidistilled water, pH 5.0.

The spectroscopy was performed to confirm the absorbance of the sample. The results confirmed the absorbance of the fluorochrome conjugated with PLGA in the near-infrared region, generating an emission peak >700 nm (Figure 2).

### 3.2. In Vitro Binding of PLGA-NPs and VEGF-PLGA-NPs to KDR-Fc

**Figure 3:**
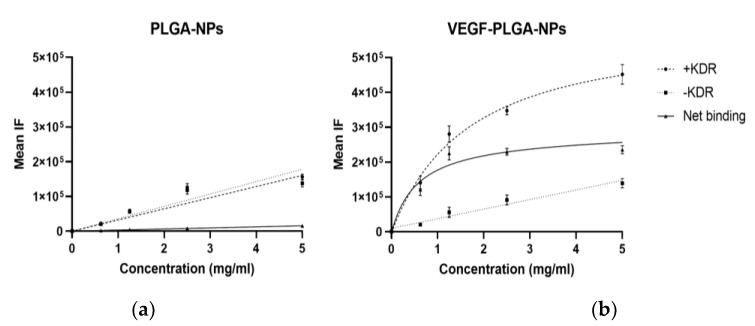
In vitro binding assay of PLGA-NPs (**a**) and VEGF-PLGA-NPs (**b**) to VEGF Receptor-2 (KDR)/Fc chimera human (KDR-Fc). KDR-Fc at concentration of 0.002 µg/µL was coated on the surface of 96-well plates. Two-fold dilutions of PLGA-NPs and VEGF-PLGA-NPs were incubated overnight at 4 °C. The mean fluorescent intensity (IF) was calculated for each well using in vivo FX station Kodak. Net binding was calculated by subtracting the mean IF in - KDR wells (negative control) to the mean IF calculated in +KDR well. One well for each dilution has not been coated with KDR-Fc and it was used as a negative control (-KDR). Results are presented as the means ± S.D (bars) of three separate experiments. Statistical analysis by Mann–Witney test showed significant difference between the two binding curves (*p* < 0.0001).

In vitro binding studies with PLGA-NPs and VEGF-PLGA-NPs are summarized in Figure 3.

PLGA-NPs showed low NET binding to KDR-Fc that increased linearly with the concentration, properly due to non-specific interactions.

On the other hand, NET binding of VEGF-PLGA-NPs reached a plateau at 1.2 mg/mL due to a VEGFR saturation, demonstrating the specific interaction between VEGF and KDR-Fc.

The results indicated the binding specificity of VEGF functionalized PLGA-NPs with the KDR-Fc.

### 3.3. In Vivo Studies

#### Pharmacokinetic and Tumor Targeting of PLGA-NPs

**Figure 4:**
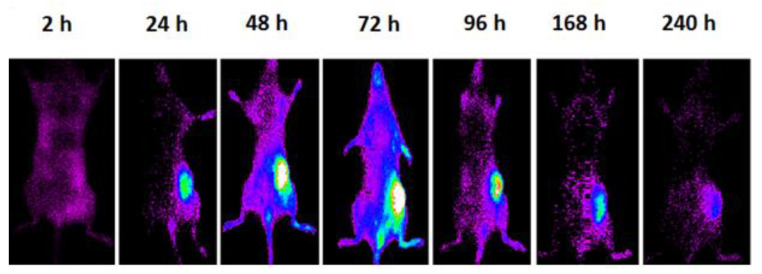
Whole body optical images of the same mouse bearing a subcutaneous syngeneic tumor at 2, 24, 48, 72, 96, 168, 240 h post-injection of 500 µg of fluorescent PLGA-NPs subcutaneously in the right flank.

**Figure 5:**
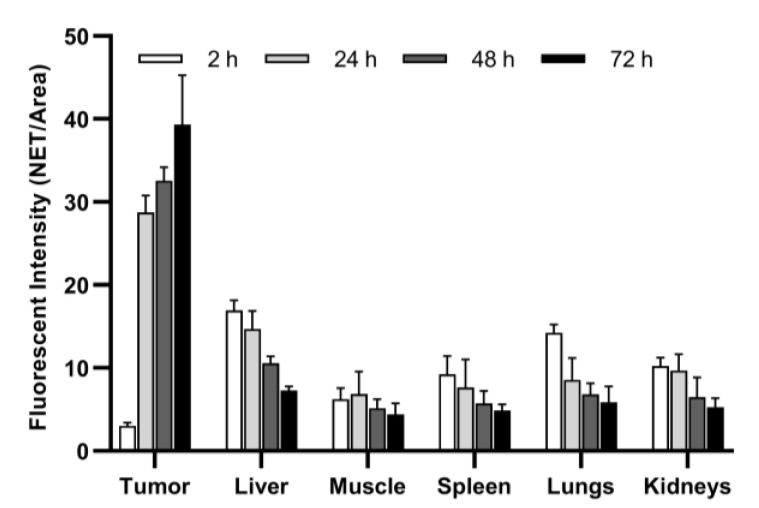
Biodistribution of PLGA-NPs in BALB/c mice. Data are expressed as average fluorescence (NET/Area) ± SD of five different mice per time point.

In vivo pharmacokinetic studies of PLGA-NPs showed maximum tumor uptake at 72 h p.i., as shown in Figure 4. This result was confirmed by ex vivo imaging of the collected organs and a semi-quantitative analysis of the ROIs (Figure 5, Table 2). After PLGA-NPs injection, the tumor was clearly visible in planar whole body images, with a signal that increased up to 24 h with a high contrast to noise ratio. Tumor accumulation of PLGA-NPs gradually decreased with time over 240 h p.i.

Ex vivo studies (Table 2) revealed that the main route of excretion is the liver due to the size of PLGA-NPs that exceed the glomerular filtration cut-off. However, fluorescence from the kidneys was also observed, probably due to the elimination of PLGA metabolites. In the blood circulation, PLGA are cleared by the cells of the mononuclear phagocytic system (MPS), that are also present in lungs, thus explaining their mean IF.

**Figure 6:**
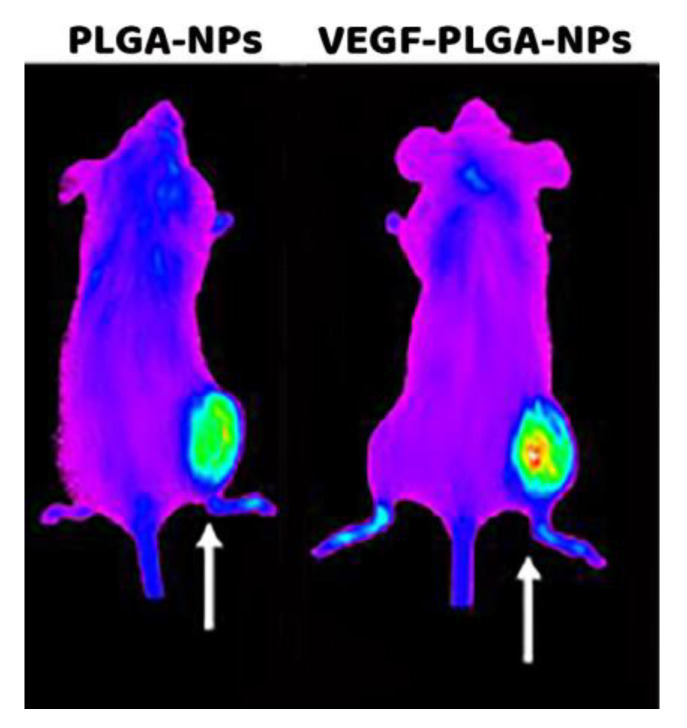
Whole body images of two mice bearing a syngeneic J744a.1 tumor in the right thigh and acquired 24 h post-injection (p.i.) of native PLGA-NPs (left) and VEGF-PLGA-NPs (right).

**Figure 7:**
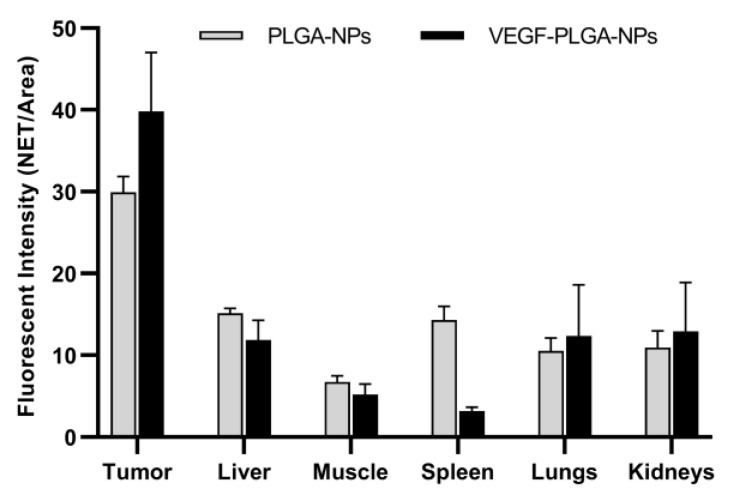
Comparative distribution of PLGA-NPs and VEGF-PLGA-NPs at 24 h post-injection (p.i.) in collected organs. Data are expressed as average fluorescence (NET/Area) ± SD of five different mice per group.

The signal from the spleen, lungs, liver, and kidneys decreases from 2 h to 24 h, whereas the signal from the tumor increases with time. Imaging studies with PLGA-NPs and VEGF-PLGA-NPs are reported in Figure 6. Mice injected with VEGF-PLGA-NPs showed increased tumor uptake and higher T/M ratio if compared to PLGA-NPs (Figure 7, Table 3).

## 4. Discussion

Recently, biodegradable PLGA-NPs have been intensively investigated as carriers for drugs, peptides, and other molecules to treat cancer with low systemic toxicity [21,22]. However, nanoparticles are versatile molecules that could be also used for diagnostic imaging [23].

PLGA-NPs characteristics such as size, surface charge, and polymer composition, can be tuned to modify their in vivo biodistribution and make them suitable tools for different purposes [24,25].

For example, they could be even modified to enhance binding and active targeting to specific tumor antigens [26,27]. Given the many reports on the use of PLGA-NPs as a delivery system, we wanted to test the potential of specifically designed (lactic acid:glycolic acid ratio of 50:50, average size of 100–200 nm) NIR-fluorescent PLGA-NPs as theranostic tools for diagnosis and therapy of cancer. Preliminary results obtained by our group and confirmed by this study, showed that PLGA-NPs have suitable characteristics to be used as an in vivo targeting tool due to high accumulation in tumors thanks to the EPR effect. Indeed, high T/B ratios in tumors are achieved within 24 h p.i. of NIR-PLGA-NPs and reach their maximum at 72 h. To increase their accumulation in tumor lesions and reduce uptake in the liver and kidneys, we also developed fluorescent VEGF-conjugated NPs. Indeed, pathological neo-angiogenesis is involved in tumor growth and distant metastatization [28]. The angiogenic cytokines, as the vascular endothelial growth factor A (VEGF-A), are involved in the growth and remodeling of vessels in the tumor microenvironment [29,30,31]. Several targeted therapies based on VEGF/VEGFR signaling have been developed in different oncological diseases [32]. For example, the clinically approved anti-VEGF monoclonal antibody (mAb), bevacizumab, recognizes the free VEGF isoforms blocking their binding with VEGFR [33]. The anti-angiogenic tyrosine kinase inhibitors (TKIs), sorafenib, and sunitinib, were approved to target the VEGFR2, blocking the signaling cascade [34].

The clinical implications of VEGF-targeted therapies caused several benefits for the majority of patients, with the exception of a small fraction [35]. This highlighted the importance of angiogenic markers when it comes to theranostic. In literature PLGA-NPs are widely described as a delivery system, encapsulating inside the polymers drugs or molecules usually with the double emulsion-solvent evaporation technique or nanoprecipitation method [36,37,38]. VEGF molecules are usually encapsulated inside the PLGA-NPs for therapeutic purposes as therapeutic angiogenesis or tissue regeneration [39,40]. In the present study, we functionalized the surface of NIR-fluorescent PLGA-NPs with the rhVEGF-A165 analog to enhance their accumulation in tumors.

A similar approach has been described by Shi et al. that used recombinant human VEGF-C and achieved successful conjugation of the protein with NPs. However, their particle size was bigger than our (400 nm vs. 150 nm) and no biodistribution in vivo was shown [41].

Our results from DLS analysis showed a significant drop in the zeta potential from −37.6 mV (of PLGA-NPs) to −9.4 mV (of VEGF-PLGA-NPs). However, the zeta potential indicates the potential difference across the boundaries between liquid and solid phases. This value should be higher than +25 mV or lower than −25 mV to indicate good stability. In the range between +25mV and −25 mV it indicates poor or no stability. The zeta potential should be evaluated together with the PDI that shows the dispersity of NPs in the liquid suspension and should be closer to 0. This index reveals the degree of dispersion of NPs in suspension (PDI higher than 0.7 indicates polydisperse NPs and aggregates; PDI less than 0.5 indicates monodisperse NPs without aggregates) [42]. Our results showed that VEGF-NPs, despite a suboptimal zeta potential (−9.4 mV), have an excellent value of PDI (0.17) and therefore reasonably stable to be used for in vitro or in vivo studies.

In vitro binding studies to KDR-Fc, showed that, despite some non-specific interactions with the plastic surface, the binding of VEGF-PLGA-NPs to VEGF receptors (KDR-Fc) is specific. This result supports the finding of an increased T/M ratio of VEGF-PLGA-NPs if compared to PLGA-NPs. We also observed in vivo a lower uptake in other major organs (e.g., liver and spleen) and higher accumulation in kidneys. From a translational point of view, it would be very useful to have a diagnostic imaging probe that allows us to evaluate the degree of accumulation in tumors prior to administer the same NPs containing an anticancer drug. Fluorescent probes, despite their usefulness in pre-clinical applications, have limited penetration in tissues and are not suitable for human studies [43]. The limited penetration of light can be overcome by the use of radioactive isotopes, such as Copper-64 (T_1/2_ = 12.7 h) for positron emission tomography (PET) or Technetium-99 m (T_1/2_ = 6 h) for gamma-camera imaging [44]. Our study, showing high tumor accumulation of PLGA-NPs (with or without VEGF) within 24 h from injection, is preparatory for the development of radiolabeled NPs with diagnostic and/or therapeutic purposes, replacing the NIR-fluorescent probe. The use of radioisotopes, especially alfa or beta- emitters, poses a serious problem of liver and kidneys radiotoxicity and we believe that the added value of VEGF (or other targeting molecules) functionalization might mitigate this issue [45,46]. In this perspective, we selected for targeting studies the time point of 24 h p.i., as it matches with the half-life of most common diagnostic isotopes. Indeed, it would be of great interest to investigate later time points with VEGF-NPs, especially if radiolabeled with a therapeutic isotope, but priority should be given to test NPs radiolabeled with diagnostic isotopes to confirm the results obtained with optical imaging.

## 5. Conclusions

The use of PLGA as a delivery system for several drugs has already been approved by the FDA and several studies have focused on their design for this purpose. Despite the extensive work with the PLGA in the therapeutic field, they have not been extensively explored as an imaging tool in humans [47,48]. Despite the recent progress in nanomedicine, the imaging depth-limit of fluorescence does not allow the application of these NPs for human diagnostic purposes [49]. For this reason, our strategy was to use fluorescent-PLGA-NPs as screening probes to assess pharmacokinetic, tumor targeting, and T/M ratio of native and functionalized PLGA-NPs. Second step will be to develop radiolabeled NPs with translational potential.

In summary, our study confirms the potential of 50:50 100–200 nm PLGA-NPs as a theranostic tool in oncology. Functionalization with targeting molecules, such as VEGF, can increase their T/M ratio in vivo, but the replacement of fluorescent probes is mandatory to translate results in humans.

## Figures and Tables

**Table 1:** Characterization of PLGA-NPs and VEGF-PLGA-NPs to the DLS. Data are expressed as mean ± SD of three measurements.

	PLGA-NPs Mean ± SD	VEGF-PLGA-NPs Mean ± SD	*t* Test (*p*)
**Zeta average (nm)**	180.2 ± 17.08	173.03 ± 7.39	n.s.
**Polydispersity index**	0.25 ± 0.02	0.17 ± 0.01	**0.01**
**Mean intensity (nm)**	169.73 ± 15.10	208.60 ± 4.97	**0.03**
**Zeta potential (mV)**	−37.6 ± 0.67	−9.43 ± 0.25	**0.0001**

Bold: statistically significant (*p* < 0.05).

**Table 2:** Ex vivo fluorescence (NET/Area) of organs at different time points.

*Parameter*	2 h	24 h	48 h	72 h	*p*
	Mean ± SD (95% CI)	Mean ± SD (95% CI)	Mean ± SD (95% CI)	Mean ± SD (95% CI)	
Tumor *	3.03 ± 0.37 (2.58 to 3.49)	28.75 ± 2.02 (26.23 to 31.26)	32.58 ± 1.62 (30.57 to 34.59)	39.32 ± 5.95 (31.92 to 46.71)	**<0.0001**
Liver **	16.94 ± 1.19 (15.47 to 18.41)	14.67 ± 2.19 (11.94 to 17.39)	10.56 ± 0.85 (9.50 to 11.61)	7.26 ± 0.51 (6.62 to 7.89)	**<0.0001**
Muscle **	6.23 ± 1.34 (4.57 to 7.89)	6.83 ± 2.72 (3.46 to 10.21)	5.16 ± 1.05 (3.86 to 6.46)	4.39 ± 1.34 (2.73 to 6.05)	n.s.
Spleen	9.25 ± 2.19 (6.53 to 11.96)	7.64 ± 3.38 (3.44 to 11.84)	5.73 ± 1.52 (3.84 to 7.62)	4.84 ± 0.75 (3.90 to 5.77)	**0.02**
Lungs	14.22 ± 1.01 (12.96 to 15.48)	8.56 ± 2.64 (5.28 to 11.85)	6.83 ± 1.29 (5.22 to 8.44)	5.86 ± 1.92 (3.47 to 8.25)	**<0.0001**
Kidneys	10.21 ± 1.01 (8.96 to 11.46)	9.70 ± 1.96 (7.26 to 12.14)	6.49 ± 2.35 (3.58 to 9.40)	5.28 ± 1.08 (3.93 to 6.62)	**0.0006**

* log_10_ transformed; ** quadratic transformed; Tumor: post-hoc analysis: *p* (2 h vs. 24 h) **< 0.0001**; *p* (2 h vs. 48 h) **< 0.0001**; *p* (2 h vs. 72 h) **< 0.0001**; *p* (24 h vs. 72 h) = **0.0023;** Liver: post-hoc analysis: *p* (2 h vs. 24 h) = **0.042**; *p* (2 h vs. 48 h) **< 0.0001**; *p* (2 h vs. 72 h) **< 0.0001**; *p* (24 h vs. 48 h) = **0.0016**; *p* (24 h vs. 72 h) **< 0.0001**; Spleen: post-hoc analysis: *p* (2 h vs. 72 h) = **0.026**; Lungs: post-hoc analysis: *p* (2 h vs. 24 h) = **0.0008**; *p* (2 h vs. 48 h) **< 0.0001**; *p* (2 h vs. 72 h) **< 0.0001**; Kidneys: post-hoc analysis: *p* (2 h vs. 48 h) = **0.015**; *p* (2 h vs. 72 h) = **0.0015**; *p* (24 h vs. 48 h) = **0.039**; *p* (24 h vs. 72 h) = **0.004**. Bold: statistically significant (*p* < 0.05).

**Table 3:** fluorescence (NET/Area) of different organs at 24 h p.i. of PLGA-NPs and VEGF-PLGA-NPs.

*Parameter*	PLGA-NPs	VEGF-PLGA-NPs	*t* Test (*p*)
	Mean ± SD (95% CI)	Mean ± SD (95% CI)	
Tumor °	29.95 ± 1.92 (27.56 to 32.33)	39.83 ± 7.17 (30.92 to 48.74)	**0.03**
Liver °	15.17 ± 0.55 (14.48 to 15.85)	11.86 ± 2.42 (5.84 to 17.88)	n.s.
Muscle	6.73 ± 0.73 (5.82 to 7.65)	5.18 ± 1.31 (3.56 to 6.80)	n.s.
Spleen °	14.29 ± 1.71 (12.18 to 16.41)	3.20 ± 0.44 (2.50 to 3.89)	**<0.0001**
Lungs °	10.53 ± 1.59 (8.55 to 12.50)	12.37 ± 6.24 (4.62 to 20.12)	n.s.
Kidneys	10.96 ± 2.03 (8.43 to 13.49)	12.92 ± 5.99 (5.48 to 20.36)	n.s.
T/M *	4.49 ± 0.54 (3.81 to 5.17)	7.90 ± 1.61 (5.90 to 9.90)	**0.0003**

* Inverse transformed; ° Satterthwaite correction. Bold: statistically significant (*p* < 0.05).
